# Foraging Behavior under Starvation Conditions Is Altered via Photosynthesis by the Marine Gastropod, *Elysia clarki*

**DOI:** 10.1371/journal.pone.0022162

**Published:** 2011-07-20

**Authors:** Michael L. Middlebrooks, Sidney K. Pierce, Susan S. Bell

**Affiliations:** Department of Integrative Biology, University of South Florida, Tampa, Florida, United States of America; Institute of Marine Research, Norway

## Abstract

It has been well documented that nutritional state can influence the foraging behavior of animals. However, photosynthetic animals, those capable of both heterotrophy and symbiotic photosynthesis, may have a delayed behavioral response due to their ability to photosynthesize. To test this hypothesis we subjected groups of the kleptoplastic sea slug, *Elysia clarki*, to a gradient of starvation treatments of 4, 8, and 12 weeks plus a satiated control. Compared to the control group, slugs starved 8 and 12 weeks displayed a significant increase in the proportion of slugs feeding and a significant decrease in photosynthetic capability, as measured in maximum quantum yield and [chl a]. The 4 week group, however, showed no significant difference in feeding behavior or in the metrics of photosynthesis compared to the control. This suggests that photosynthesis in *E. clarki*, thought to be linked to horizontally-transferred algal genes, delays a behavioral response to starvation. This is the first demonstration of a link between photosynthetic capability in an animal and a modification of foraging behavior under conditions of starvation.

## Introduction

Foraging behavior of animals, defined here as actively searching for and consuming food, encompasses diverse processes such as dispersal, predator-prey interactions, predation risk, and resource optimization [1], [2], [3], [4]. Animals deprived of food are more likely to travel further and invest more time in search of food than satiated conspecifics [5], thereby utilizing energy that might otherwise have been allocated to purposes such as growth or reproduction. Additionally, starved animals may exhibit reduced anti-predatory behavior to gain increased access to a food source [3]. Some models based upon risk-sensitive foraging theory suggest that animals will choose a more variable array of energy-yielding food items during periods of starvation [4], [6]. Specific behavioral displays in response to starvation can be highly variable, as environmental conditions (e.g. predators, refuge) [7] or physiological traits, such as fat storage [8],[9], might modulate responses. Altogether, starvation generally modifies the actions related to food acquisition [3], [8], [10], [11] which in turn may have consequences for an organism's survival and interspecific interactions [3].

Most animals which forage are limited to acquiring energy by ingestion [12]. However, a number of species from diverse taxa not only acquire energy through assimilation of ingested food but also by photosynthesis conducted by endosymbiotic zooxanthellae or stored algal chloroplasts and are termed mixotrophic [12], . Depending on light conditions, these photosynthetic animals can derive a significant portion of energy yielding metabolites via photosynthetic pathways [15]. As a result, photosynthetic animals may have different behavioral responses to a lack of food than are displayed by non-photosynthetic capable species. Thus, despite food limitation, if photosynthesis provides sufficient metabolites, then the incidence of food searching behavior, and the associated elevated risk of mortality, might be correspondingly reduced or even eliminated. Although studies have demonstrated the effect of photosynthesis on feeding rates in corals [16], [17], the foraging behavior of starved photosynthetic-capable animals which are motile has not been well studied.

**Figure 1:**
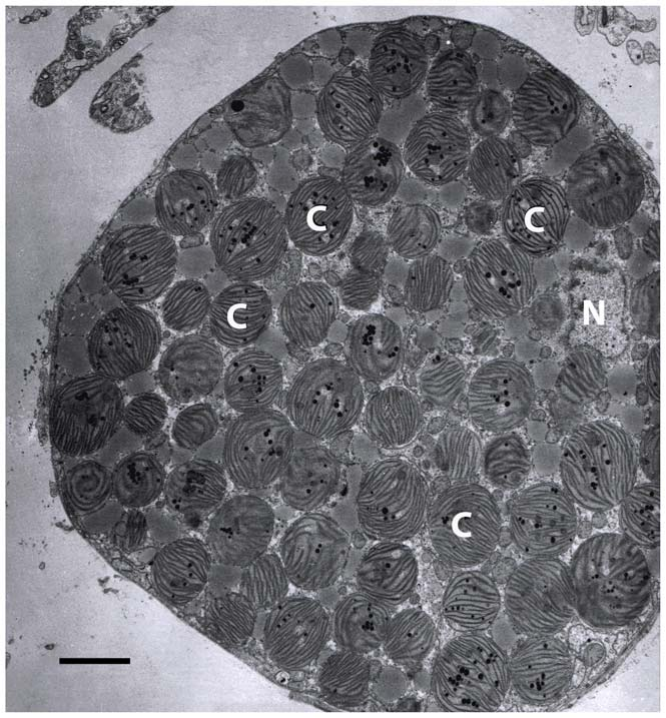
Electron micrograph of a digestive tubule cell of *E. clarki*. The digestive tubule cell is densely packed with sequestered chloroplasts. C = chloroplast, N = nucleus. Scale bar represents 3 µm. Image taken by Nicholas Curtis.

Most animal species capable of utilizing photosynthesis as an energy source are aquatic and sessile (e.g. corals, sponges, giant clams) or have limited motility (e.g. benthic jellyfish), and thereby do not forage at all [12]. However, the sacoglossan (Opisthobranchia: Mollusca) sea slugs are highly motile and actively forage on algae, which is usually siphonaceous and typically found in shallow water [18]. Importantly, many sacoglossans are capable of kleptoplasty, a process by which slugs photosynthesize using chloroplasts which are sequestered from the algal food by specialized cells of the digestive tubules (Figure 1) [13], [18], [19], [20]. Nuclear genes horizontally transferred from algae into the slugs likely play an important role in the slugs' ability to photosynthesize [21], [22]. These combined features suggest that kleptoplastic sea slugs are a specialized group of herbivores which can be utilized to examine how the level of satiation/starvation affects foraging behavior in a photosynthetic animal. Although increased foraging efforts under starvation conditions and a decrease during satiation is usual for many species [2], [5], the foraging behavior of photosynthetic sea slugs may be different, if food is withheld, provided that photosynthesis continues.

Previous studies of kleptoplasticslugs have considered the evolutionary benefit of supplemental energy provided by sequestered chloroplasts. A relatively large amount of sequestered chloroplasts could allow less time to be spent foraging [23]. Kleptoplasty might also increase slug survival during times of famine [24]. However, among sacoglossan species, the length of time that algal chloroplasts remain functional is quite variable, ranging from only several hours to up to 9 months [18], [25]. Therefore the impact of photosynthetic metabolites must also vary among species, and kleptoplasticslugs experiencing food limitations may only manage to delay behavioral changes related to food gathering, rather than completely avoid modifying their behavior. Although kleptoplasty provides metabolites to slugs, ultimately most slug species require ingested food at least to replace degraded chloroplasts [18], [25] with the notable exception of *Elysia chlorotica* which can complete the entire adult portion of its life cycle (up to 9 months) relying only on photosynthesis [26], [27]. Thus, the benefits of kleptoplasty are time limited. It is likely that, for most kleptoplastic organisms, as photosynthetic function declines foraging behavior will increase.

We describe here a set of experiments examining the incidence of foraging behavior displayed by a kleptoplastic slug subjected to different conditions of starvation. Our hypothesis was that starved slugs would not change their foraging behavior while able to photosynthesize. However, as starvation continues and photosynthetic activity decreases due to chloroplast failure [18], [25] foraging behavior likely is triggered. Therefore once photosynthesis has ceased, an increase in starvation time should also increase the probability of slugs exhibiting behavior linked to food acquisition.

## Methods

### Source and Maintenance of Slugs

**Figure 2:**
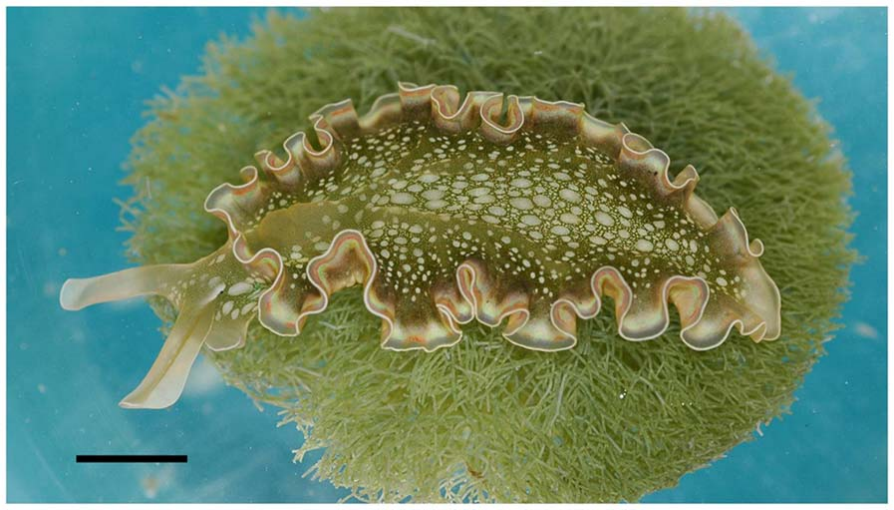
*Elysia clarki* on the algal food source, *Penicilluscapitatus*. Photo reprinted with permission from Curtis et al. (2006) [30] Scale bar represents 500 mm.

*Elysia clarki* (Figure 2), a sacoglossan species [28], is an excellent organism for evaluating the relationship between kleptoplasty and feeding behavior. The slug lives in the Florida Keys in near-shore, low wave energy habitats, such as mangrove swamps, borrow pits, and mooring canals. It feeds suctorially on several species of siphonaceous green algae including *Penicillus capitatus*, *P. lamourouxii*, *Halimeda incrassata*, *Bryopsis plumosa*, and *Derbesia tenissima* [29], [30], [31]. The slug sequesters the chloroplasts from all of these species and uses them for photosynthesis. Furthermore, *E. clarki* photosynthesizes using the stored chloroplasts for up to three months without ingesting food and attains relatively large size (up to 35 mm) [28]. Unlike the case for many *Elysia* species, which are tiny, observations of feeding behavior and measurements of photosynthesis are possible with *E. clarki*.

### Project Overview

In order to assess the effects of starvation on photosynthesis and feeding behavior of *E. clarki*, slugs were collected from the field and assigned to feeding trials representing different levels of provided food. Once desired time of starvation for the experiment had been established, the slugs' feeding behavior was assessed. Finally, the amount of sequestered chlorophyll and photosynthetic rates of the slugs were measured and any display of feeding behavior was noted to determine whether photosynthesis was associated with delayed onset of foraging.

Specimens of *E. clarki* approximately 2cm in length were collected by snorkel from a borrow pit on Grassy Key, FL (24°44′56.07″N, 80°58′42.77″W) with permission from the Florida Fish and Wildlife Conservation Commission (permit # SAL-11-0616-SR) and then transported to the laboratory at the University of South Florida in Tampa, FL during July 2009. Twenty-four groups of 4 slugs were randomly assigned to 10 L aquaria filled with artificial seawater (Instant Ocean™) and treated one time before the start of the experiments with the antibiotics, Penicillin and Streptomycin (100 µg/ml) to control bacteria. Aquaria were maintained at room temperature (∼20°C). Photoperiod was maintained by alternating 12 hr light/dark cycles provided by overhead cool-white fluorescent lights. All slugs were initially starved for 4 weeks to ensure empty guts before the start of the experiment. Then, for a 2 week period, all aquaria were stocked with the algae *P. capitatus* (Figure 2), collected from a local seagrass bed near Tarpon Springs, FL (28°8′38.73″N, 82°47′26.49″W), and slugs were allowed to feed *ab libitum*.

After the 2 week feeding period, slugs were randomly assigned to one of three starvation level treatments, or a satiation control. The experiment was designed to represent a gradient of food availability and associated physiological state ranging from no food (starvation) to continuous access to food (satiation). Completely starved slugs had all algal food removed at the start, and were starved for the entire 12 week trial. Other slugs received food for 4 or 8 weeks respectively, and were then starved for the rest of the 12 week trial. The control group received a continuous supply of food for the entire 12 week experiment and slugs in this treatment were considered satiated. Each of the feeding treatments was replicated in 6 aquaria (n = 24 total). All slugs were provided light (overhead cool white fluorescent lights) on the 12 hr light/dark cycle to allow photosynthesis. After 12 weeks, the slugs from each feeding treatment were tested for their response to food.

### Feeding Behavior

In order to examine the impact of starvation on feeding behavior of *E. clarki*, we tested feeding behavior of slugs as follows: Observations of slugs were made in 2L glass beakers filled with 1L glass beakers filled with 1L of artificial seawater (Instant Ocean™), and each containing an individual cap and stipe of *P. capitatus*, placed on the bottom. *Penicillus capitatus* was the only food source presented to slugs during the trials. The beakers were maintained in ambient light conditions during the feeding trials which took place during daylight hours. Individuals of *P. capitatus* were only used once and beakers were then emptied and cleaned for subsequent trials. Individual slugs were placed into containers with their anterior end ∼1cm from the alga to ensure slugs would have quick access to the food. Preliminary trials determined that this design represented the most effective arrangement to determine whether a slug initiated the feeding response.

After placement into an experimental container, slugs were observed for 5 min to determine if any feeding behavior occurred, defined as moving to and remaining on the alga. Although the physical presence of the slug on the alga does not necessarily indicate feeding, it reflects the position of the slug when it naturally feeds in the field. Because the slugs feed by sucking contents out of algal filaments, the densely packed architecture of filaments [32] prevents actual confirmation that the slug fed on the alga within the 5 min observation period. The length of feeding observations was constrained to 5 min because slugs were subsequently analyzed for chlorophyll concentration and additional feeding time could increase the amount of chlorophyll present in an individual slug.

To test the prediction that the proportion of slugs displaying feeding behavior (yes/no) would increase as time of starvation (continuous predictor) increased, we analyzed the results of experimental trials using the general linear model (GLM) via Statistica™. For the feeding experiment, slugs were analyzed as the of individuals that fed for each starvation treatment. To obtain a direct comparison between treatments, each possible combination of pairs of the 4 treatment groups was tested separately to evaluate differences using a Chi-squared goodness of fit test. For example, the control group and the 12 week starvation group were analyzed together without the 4 and 8 week starvation groups.

### Changes in Photosynthetic Ability

Once behavior had been evaluated, we measured two aspects of photosynthetic capabilities of sequestered chloroplasts within the slugs used in behavioral observation: Pulse Amplitude Modulated (PAM) Fluorometry for quantifying chlorophyll fluorescence and chlorophyll a (Chl a) extraction to determine the amount of Chl a remaining in the slugs. We used both fluorescence and Chl a concentration [Chl a] because each provides a different measure of photosynthesis. The amount of Chl a serves as an estimate of the amount of sequestered chloroplasts within a slug [24], [33], [34], while fluorescence measures the photosynthetic activity [35]. The latter may or may not be related to the amount of algal chloroplasts depending on the condition of the chloroplasts. Therefore, although photosynthetic activity and [Chl a] are often correlated, high [Chl a] can be present despite low photosynthetic activity [13].

### Chl a and PAM Fluorescence

Photosynthetic activity of each slug used in the feeding trial was measured using a PAM Fluorometer, which measures chlorophyll a fluorescence originating from photosystem II by emitting a strong pulse of light and measuring the returning fluorescence [35], [36] (Diving PAM, Walz, Germany). PAM has also been used successfully by others to measure photosynthesis in sea slugs [35]. Slugs were first dark adapted for 20 min in a dark room, and then measured for maximum quantum yield of fluorescence (ϕ_IIe_) using the PAM and the following equation:
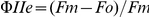
where F_m_ is the maximum fluorescence during the light pulse and F_0_ is the fluorescence measured in dark-acclimated tissues before the light pulse is emitted [35]. Each slug was measured three times to ensure an accurate reading of ϕ_IIe_ and a mean value of all three ϕ_IIe_ readings was determined.

### Chl a measurement

Upon completion of the PAM measurements, slugs were freeze-dried and weighed (g). The freeze-dried slugs were each homogenized in acetone and then centrifuged (∼12,000× G). The supernatant was saved and absorbance determined at 423 nm (Beckman Coulter DU 640™ Spectrophotometer), the wavelength at which Chl a absorbs [37]. [Chl a] were then calculated from a standard Chl a curve and normalized (µg chl a/g dry weight of slug) [37].

*A. priori* we expected to find higher maximum quantum yield and [Chl a] in slugs which had been feeding more recently. Maximum quantum yield was analyzed across treatments using a one way analysis of variance (ANOVA) followed by a Tukey HSD *post hoc* test with starvation length as the predictor and ϕ_IIe_ as the dependent variable. [Chl a] was analyzed across starvation treatments using a one way ANOVA and Tukey HSD *post hoc* analysis after being log-transformed to meet ANOVA assumptions of homoscedasticity. For these analyses, starvation length was the predictor and [Chl a] the dependent variable.

## Results

**Figure 3:**
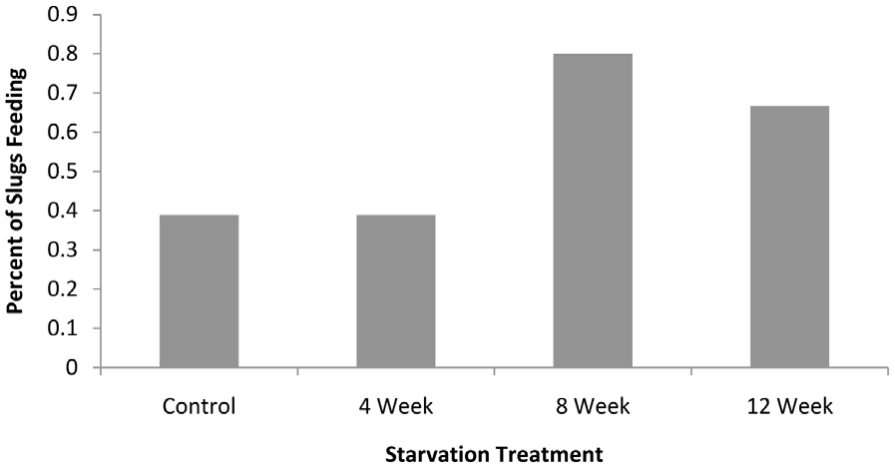
Percentage of slugs displaying feeding behavior after each starvation period. Data represent all slugs tested for each starvation treatment group.

Significant differences in slug feeding behavior and length of starvation were clearly evident. Slugs from all treatments displayed some feeding behavior; however, slugs from the continuously fed control, as well as those starved for 4 weeks, were less likely to feed than slugs starved for 8 and 12 weeks (Figure 3). Thirty-three percent of slugs in both the control and 4 week starvation group displayed feeding behavior, compared to 73% and 69% of the slugs in the 8 and 12 week starvation period, respectively. The length of time that slugs were starved was significantly associatedwith the proportion of slugs displaying feeding behavior (p<0.001, F = 13.67) (GLM). Pair-wise Chi-squared goodness of fit tests indicated that slug foraging behavior in both the control and 4 week starvation treatments were not significantly different from each other, but both were different from slugs in the 8 (p<0.05, χ^2^ = 4.311) and the 12 (p<0.05, χ^2^ = 4.200) week starvation groups.

**Figure 4:**
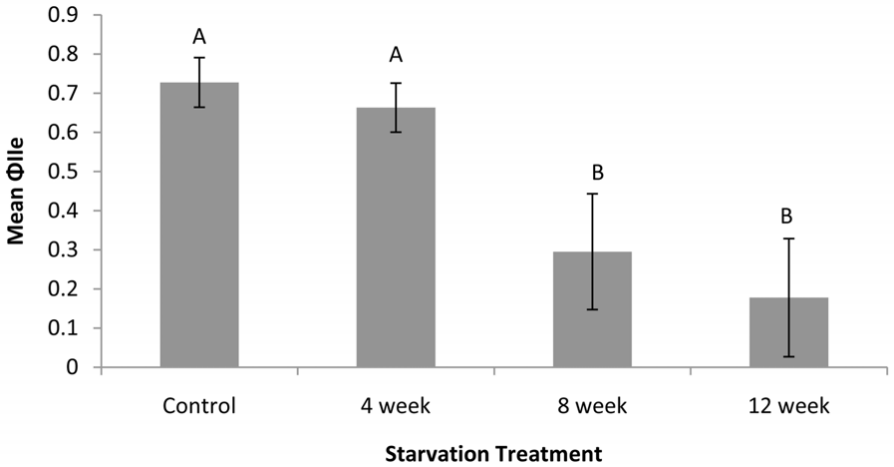
Mean maximum quantum yield (+/− standard deviation) for slugs from each starvation treatment. Letters indicate significant differences among treatments.

The length of starvation significantly reduced Chl a fluorescence (PAM) (F_3,9_ = 33.81, p<0.001). The mean ϕ_IIe_ in the continually feeding control slugs was approximately 2.5× higher than slugs starved for 8 weeks and over 3× higher than slugs starved for 12 weeks. Both the controls and slugs from the 4 week starvation group had a significantly higher mean ϕ_IIe_ than that of slugs starved for either 8 or 12 weeks (p<0.001) (Figure 4).

**Figure 5:**
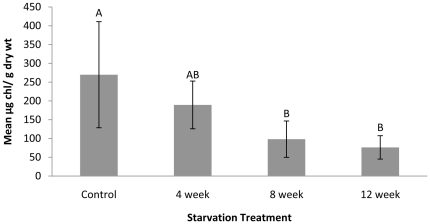
Mean [Chl a] (+/− standard deviation) for slugs for each starvation treatment. Letters indicate significant differences among treatments. Presented data were back-transformed after a logarithmic transformation to meet assumption of homoscedasticity.

[Chl a] (µg chla/g dry weight of slug) in slugs declined as the length of starvation increased. After 12 weeks of starvation, [Chl a] was less than 1/3 that recorded for control slugs. An overall decrease in [Chl a] was detected in all starvation groups (F_3,9_ = 10.10, p<0.001) (Figure 5). Tukey HSD *post hoc* analysis showed that [Chl a] from both the 8 and 12 week starvation treatments were significantly lower than that for the continuously fed control (p<0.01) (Figure 5). The [Chl a] in slugs starved for 4 weeks was only significantly different from the slugs starved for 12 week (p<0.01).

## Discussion

Eventually, an increased length of starvation increased the likelihood of foraging behavior in *E. clarki*, asusual with starvation [2], [3], [11]. However unlike earlier studies, our findings highlight that *E. clarki's* foraging behavior is further modified by its photosynthetic capability. The onset of the foraging behavior in *E. clarki* in response to prolonged starvation was delayed during the time period that the slugs' photosynthetic capabilities remain relatively high. Some slugs foraged although satiated, under conditions of a continuous food supply, so foraging is not eliminated for all members of the population. However, the incidence of foraging behavior increased as a decrease in photosynthetic ability occurred. In effect, the presence of kleptoplasts and their photosynthetic ability allow *E. clarki* to behave, even after a month of starvation, in a similar way to slugs that have been satiated. This is the first report to demonstrate the role of photosynthesis in modifying the foraging behavior in a starved mixotrophic animal.

Although reducing movement to conserve energy in times of famine or starvation might seem like a viable strategy, many species actually increase foraging effort under starvation conditions [5], [38]. Nutritional state has long been implicated in behavioral changes of starved animals traveling more often and farther distances in search of food than satiated animals [5]. For example, starvednymphs of *Podisus nigrispinus* are more likely to disperse and move further from a starting location than satiated nymphs [5]. Other species such as the sea star, *Leptasterias polaris*, are more likely to move when starved, and are also more likely to orient towards a water current, increasing the likelihood of encountering prey, than satiated conspecifics [39]. Behavioral changes, such as consuming chemically-defended food that typically would have been avoided, have also been documented in starved herbivorous urchins [40], [41]. If our laboratory study predicts field behavior, then a large percentage of the population of *E. clarki* likely exhibits a delay of weeks after food sources become scarce before at least some animals display a behavioral change and commence foraging. Moreover, by not feeding slugs can remain located in a suitable habitat, in regards to conditions such a light availability and wave energy. Reduced feeding activity by slugs which photosynthesize may also allowthe algal food resources sufficient relief from grazing pressure to regrow.

One other consequence of starvation is that it may induce behavioral changes in an animal thatincrease the risk of predation. For example, the whelk, *Acanthina monodon*, normally reduces feeding in the presence of a predator, but when starved, will continue to feed despite the proximity of the predator [42]. The hermit crab, *Dardanus pedunculatus*, hosts symbiotic sea anemones on its shell which protects the crab from predation. However, when starved, the crab will eat its anemones, decreasing its camouflage and correspondingly increasing the risk of predation [43]. Although *E. clarki* is a relatively cryptic species, delayed foraging may still provide benefit from reduced exposure to predation as foraging behavior becomes less likely. While not specifically examined in *E. clarki*, predation has been demonstrated on several other cryptic sacoglossan species [44]. However, the advantage of avoiding predation in relation to foraging may be difficult to demonstrate in sacoglossans because the sequestered chloroplasts that provide metabolites also modify the slugs' color and serve as a camouflage [45].

Although the ability to photosynthesize delays foraging changes in slugs of *E. clarki* starved 8 to 12 weeks, it is not clear if similar patterns occur in other photosynthetic animals. Studies on corals have had mixed results. Some species show an increase in feeding when light conditions, and thus photosynthesis, are reduced [17], while others show no indication that the rate of feeding changes in relation to the condition of their symbiotic zooxanthellae [16]. However, corals are non-motile and thus do not engage in foraging behavior, as defined above, making them poor candidates for demonstrating the effects of starvation on the foraging of photosynthetic animals. It is more likely that other motile, photosynthetic slug species will behave similarly to *E. clarki*. The timing of a behavioral change will likely depend on the duration of photosynthesis in a given species. Species with short-lived photosynthesis, such as *Elysia ornata* [25], may have a rapid response to starvation with little delayed feeding while others like *E. chlorotica* [26], which never lose photosynthetic ability once symbiotic plastids are acquired, will likely exhibit little to no difference in feeding behavior between starved and fed individuals. Therefore, the ability to sustain photosynthesis, which may be related to horizontally transferred-algal genes [21], [22], is likely to determine the effect of starvation on foraging behavior.

### Conclusion

The onset of a behavioral shift coinciding with a decline in photosynthesis by *E. clarki* demonstrates an aspect of foraging behavior and starvation not previously considered. *Elysia clarki* starved for 8 or 12 weeks and displaying a reduced photosynthetic ability increased the incidence of foraging behavior compared to continuously fed controls and slugs starved for 4 weeks. Our work uniquely demonstrates that foraging behavior in starved photosynthetic animals is likely to remain unchanged while photosynthesis remains functional. However, our experiments revealed that a change in foraging behavior is more likely to occur when photosynthetic activity declines under conditions of starvation.
